# DNA methylation and histone modifications regulate SOX11 expression in lymphoid and solid cancer cells

**DOI:** 10.1186/s12885-015-1208-y

**Published:** 2015-04-12

**Authors:** Lena Nordström, Elin Andersson, Venera Kuci, Elin Gustavsson, Karolina Holm, Markus Ringnér, Per Guldberg, Sara Ek

**Affiliations:** 1 Department of Immunotechnology, CREATE Health, Lund University, Lund, Sweden; 2 Danish Cancer Society Research Center, Copenhagen, Denmark; 3 Department of Oncology and Pathology, Clinical Sciences, Lund University, Skåne University Hospital, Lund, Sweden

**Keywords:** SOX11, DNA methylation, H3K27, Epigenetic regulation

## Abstract

**Background:**

The neural transcription factor SOX11 is present at specific stages during embryo development with a very restricted expression in adult tissue, indicating precise regulation of transcription. SOX11 is strongly up-regulated in some malignancies and have a functional role in tumorgenesis. With the aim to explore differences in epigenetic regulation of SOX11 expression in normal versus neoplastic cells, we investigated methylation and histone modifications related to the SOX11 promoter and the possibility to induce re-expression using histone deacetylase (HDAC) or EZH2 inhibitors.

**Methods:**

The epigenetic regulation of *SOX11* was investigated in distinct non-malignant cell populations (n = 7) and neoplastic cell-lines (n = 42) of different cellular origins. DNA methylation was assessed using bisulfite sequencing, methylation-specific melting curve analysis, MethyLight and pyrosequencing. The presence of H3K27me3 was assessed using ChIP-qPCR. The HDAC inhibitors Vorinostat and trichostatin A were used to induce SOX11 in cell lines with no endogenous expression.

**Results:**

The *SOX11* promoter shows a low degree of methylation and strong enrichment of H3K27me3 in non-malignant differentiated cells, independent of cellular origin. Cancers of the B-cell lineage are strongly marked by *de novo* methylation at the *SOX11* promoter in SOX11 non-expressing cells, while solid cancer entities display a more varying degree of *SOX11* promoter methylation. The silencing mark H3K27me3 was generally present at the *SOX11* promoter in non-expressing cells, and an increased enrichment was observed in cancer cells with a low degree of *SOX11* methylation compared to cells with dense methylation. Finally, we demonstrate that the HDAC inhibitors (vorinostat and trichostatin A) induce SOX11 expression in cancer cells with low levels of *SOX11* methylation.

**Conclusions:**

We show that *SOX11* is strongly marked by repressive histone marks in non-malignant cells. In contrast, SOX11 regulation in neoplastic tissues is more complex involving both DNA methylation and histone modifications. The possibility to re-express SOX11 in non-methylated tissue is of clinical relevance, and was successfully achieved in cell lines with low levels of *SOX11* methylation. In breast cancer patients, methylation of the *SOX11* promoter was shown to correlate with estrogen receptor status, suggesting that SOX11 may be functionally re-expressed during treatment with HDAC inhibitors in specific patient subgroups.

**Electronic supplementary material:**

The online version of this article (doi:10.1186/s12885-015-1208-y) contains supplementary material, which is available to authorized users.

## Background

During embryonic development, cell-fate decisions and lineage commitment are regulated by both transcription factors and epigenetic mechanisms. The SOX protein family of transcription factors is known to act as important regulators of embryonic development, cellular fate determination and differentiation [1,2]. SOX11, a member of the SOXC subgroup, plays an important role in both embryonic and adult neurogenesis, and is proposed to regulate self-renewal of neuronal progenitor cells [3]. The expression of SOX11 is absent in most adult differentiated tissues, further supporting the role as a stem cell specific regulator [4]. *SOX11* has been shown to be regulated by epigenetic events in pluripotent embryonic stem cells and is marked with both activating (H3K4me3) and repressive (H3K27me3) histone marks [5]. These bivalent marks are thought to keep developmentally important genes silenced, but poised for activation during lineage commitment [6]. Bivalent histone marks are often modified during cell differentiation so that only the active or repressive marks remain [7]. In agreement with this, peripheral B-cells that lack SOX11 have been reported to be strongly marked by H3K27me3 [8]. Interestingly, it has been shown that genes marked with H3K27me3 are targets for *de novo* methylation in cancer [9]. This is supported by gene expression analysis of *de novo* methylated genes that show lack of expression already in unmethylated non-malignant tissues [10].

Aberrant regulation of SOX11 has been observed in several tumors, leading to expression of the protein or silencing through promoter DNA methylation. Up-regulation of SOX11 has been reported in malignant glioma [11], medulloblastoma [12], mantle cell lymphoma (MCL) [13], as well as subsets of Burkitt’s lymphoma [14], ovarian cancer [15] and breast cancer [16]. Aberrant promoter methylation of *SOX11* has been reported in most mature B-cell lymphomas except MCL, which express SOX11 [13] and where SOX11 has functional [17] and prognostic [18] roles. Moreover, the presence of *SOX11* promoter methylation has been shown to be significantly higher in patients with lymph node metastasis compared to patients without metastasis in nasopharyngeal carcinoma [19]. *SOX11* methylation was also used in a five-gene biomarker panel to detect bladder cancer at an early stage [20]. Thus, both *SOX11* expression and methylation pattern correlate to clinical behaviour, which is of major interest in relation to the novel use of epigenetic drugs, enabling demethylation and/or reexpression of silenced genes.

In the present study, we aimed to further investigate the epigenetic regulation of *SOX11* in non-malignant (n = 7) and neoplastic cell populations (n = 42) to possibly identify new clinical subgroups with an aberrant regulation and/or expression of *SOX11*. We show that non-malignant cells have a low degree of DNA methylation but that SOX11 is enriched with H3K27me3. In neoplastic cells, the epigenetic regulation of *SOX11* is more complex. Most B-cell lymphomas are heavily methylated in the *SOX11* promoter region while solid tumor cells show a more diverse methylation pattern. Furthermore, in breast cancer, we demonstrate a correlation between *SOX11* methylation and clinical subtype.

As the use of histone deacetylase (HDAC) inhibitors in the clinic is continuously growing, we evaluated the effect of epigenetic drugs on SOX11 expression. We show that SOX11 expression could be induced in cells with low levels of methylation by HDAC but not EZH2 inhibitors.

## Methods

### FACS sorting of non-malignant B-cell populations

Pediatric tonsils (n=6) (Lund University Hospital, Lund, Sweden) were used as the source of normal non-malignant B-cells and collected under written informed consent by parents or guardians. The use was ethically approved by the regional Lund/Malmo committee (Dnr 242/2006). The lymphocyte population was isolated by Ficoll gradient centrifugation. Viable B-cell populations were sorted based on CD markers as follows: naïve B-cells (CD3-, CD19+, IgD+, CD38-), GC B-cells (CD3-, CD19+,IgD-, CD38+) and memory B-cells (CD3, CD19+, IgD-, CD27+). FACS analysis of sorted populations confirmed a purity of >95%.

### Cell culture

Forty two cell lines with different tumor origins were used to study the epigenetic regulation of SOX11. These included mantle cell lymphoma (n=10), follicular lymphoma (n=3), diffuse large B-cell lymphoma (n=2), Burkitt’s lymphoma (n=4), epithelial ovarian cancer (n=5), breast cancer (n=8), lung cancer (n=3), glioma cancer (n=5) and neuroblastoma cell lines (n=2). Two glioma cell lines were established from patient tissues and approved by the Local Ethical Board of the University of Lund, Sweden, serial no. LU307-98. Informed consent was obtained. To protect patient anonymity, tumor samples were coded to GBM-LU60 and GBM-LU93. All cell lines were cultured at 37C° in a humidified atmosphere of 5% CO_2_. Details about cell culture media and supplier are shown in Additional file 1.

### DNA preparation and bisulfite conversion

DNA was extracted and purified using QIAamp DNA Mini Kit (Qiagen, Hilden, Germany) followed by quantification on NanoDrop (NanoDrop technologies, Wilmington, DE, USA). All samples were bisulfite treated with EZ DNA Methylation Kit (Zymo Research, Irvine, CA, USA) according to manufacturer’s protocol. Five hundred nanograms of DNA were used for each bisulfite conversion and converted samples were eluted in 20 μl buffer.

### DNA methylation analysis of FACS sorted populations of non-malignant B-cells

The CpG island adjacent to the *SOX11* transcription start site was PCR amplified with primers specific for bisulfite treated DNA and subcloned into the TOPO-TA cloning vector as previously described [17]. Sequencing of individual alleles was made at GATC Biotech (Konstanz, Germany). The sequencing files were analyzed using BiQ Analyzer software [21] http://biq-analyzer.bioinf.mpi-inf.mpg.de/index.php. Sequences with poor conversion rates (<95%) and identical clones, possibly generated in the PCR reaction, were removed. Data presentation images and methylation statistics were generated using the BDPC web server [22].

### DNA methylation microarrays of human breast cells

DNA from human mammary fibroblasts, epithelial cells, and endothelial cells, as well as mesenchymal bone marrow stem cells (ScienCell Research Laboratories, CA, USA) was analyzed. Bisulfite conversion of 500 ng genomic DNA was performed using the EZ DNA Methylation kit (Zymo Research, Orange, CA, USA) following the manufacturer’s protocol. We hybridized 200 ng in 4 μl to the Infinium HumanMethylation450K BeadChip array (Illumina, San Diego, CA). The array includes five CpG sites within the SOX11 promoter (cg07065111, cp08432727, cg08526991, cg12312988, cg13667638, see Additional file 2). Bisulfite conversion and hybridization to arrays were performed by the SCIBLU facility, Lund, Sweden. Raw intensities for methylated (M) and unmethylated (U) signal were extracted from Illumina’s GenomeStudio. Beta-values were calculated as M/(M+U). Beta-values with detection p-value > 0.05 or with less than 3 beads for a signal were set as missing values. For each sample we performed a peak-based correction of Illumina I and II chemical assays similar to et al. [23]. For both assays we smoothed the beta values (Epanechnikov smoothing kernel) to estimate unmethylated and methylated peaks, respectively; and the unmethylated peak was moved to 0 and the methylated peak to 1 using linear scaling, with beta-values in between stretched accordingly. Beta-values below 0 were set back to 0 and values above 1 were set to 1.

### Analysis of the ENCODE project data

ChIP-seq data (H3K27me3 and H3K4me3) from human mammary epithelial cells were downloaded from the ENCODE project [24]. The sequence files were visualized with the Integrative Genomics Viewer (IGV).

### Methylation-specific melting curve analysis (MS-MCA) of tumor cell-lines

Primers used in MS-MCA amplify all types of epialleles that later are discriminated during the melting stage of the analysis, enabling a qualitative description of the sample. Primers for MS-MCA [25] were designed to amplify a sequence 273 bp upstream of *SOX11* transcription start site, containing 28 CpG sites (See Additional file 2). Primers used were: 5’-TTTTAATTTTTTGTAGAAGGAG-3’ and 5’-CCTTCCAAACTACACACAA-3’. Amplification and melting analysis was carried out on LightCycler 2.0 (Roche, Basel, Switzerland) using Fast Start DNA Master SYBR Green kit (Roche). Profiles of melting curves for fully methylated and unmethylated sequence was established using in vitro methylated DNA (IVM, Millipore, Billerica, MA, USA) and whole genome amplified DNA (WGA), derived with GenomiPhi V2 DNA amplification kit (GE Healthcare, Little Chalfont, Buckinghamshire, United Kingdom), respectively. Examples of how MS-MCA results was interpreted are shown in Additional file 3.

### MethyLight Analysis of tumor cell-lines

MethyLight is a highly sensitive quantitative method amplifying highly methylated alleles. Data is normalized to a reference sample and presented as percent methylated reference (PMR). MethyLight analysis [26] of the *SOX11* promoter region was performed on Roche LightCycler 480 realtime PCR using Lightcycler 480 Probes Master Kit (Roche) with primers 5’-GGTAGGAGTTACGAGTCGGAGAGA-3 and 5’-ACTACGATCGCGACAAAAAAAAC-3’ and probe 5’-[6FAM]TCGGGTTGTTTCGATCG[MGBNFQ]-3’ [20]. The assay was validated with bisulfite-treated DNA from cell lines unmethylated for *SOX11* and non-bisulfite treated genomic DNA (human genome DNA, Roche). A dilution series of fully methylated control (in vitro methylated DNA, IVM, Millipore) were included in each reaction. A separate reaction for repetitive sequence ALUC4 [27] was performed on each sample to control for input DNA. All reactions were done in duplicate and an average value of the concentration was used to determine DNA methylation level in each sample. Percent methylated reference, PMR were calculated according to the formula: PMR= (([SOX11sample]/[ALUC4sample])/([SOX11IVM]/[ALUC4IVM])) x 100.

### Pyrosequencing

The 28 CpG sites investigated with MS-MCA were sequenced in bisulfite treated samples using the PyroMark Q24 platform (Qiagen, Hilden, Germany). One set of amplification primers (fwd primer: 5’-ATGATATTTTGATAATTAGTTGAG-3’ and rev primer: 5’-[Btn]CCTTCCAAACTACACACAA-3’) and two sequencing primers (seq primer 1: 5’-AGAGAGATTTTAATTTTTTGTAGA-3’, seq primer 2: 5’-AGTAGGAGAGAGGGGTT-3’ ) were used to cover all 28 sites. PCR was carried out in a final volume of 25 μl containing PCR buffer (Qiagen), 200 μM each of dNTP, 0.4 μM each primer and 1 U of Taq HotStarTaq DNA polymerase (Qiagen). Sequencing was performed using PyroMark Gold Q24 reagents (Qiagen). Analysis of the results was carried out with the PyroMark Q24 software (Qiagen). Results from at least two sequencing events were used to calculate the methylation level at each CpG site. In vitro methylated DNA (IVM, Millipore) and whole genome amplified DNA (WGA) derived with GenomiPhi V2 DNA amplification kit (GE Healthcare), were used as fully methylated and unmethylated control, respectively.

### RNA isolation and Real-Time qPCR assessment of SOX11

*SOX11* mRNA expression was investigated using real-time quantitative PCR. Cells were lysed and cDNA synthesized using iScriptTM Synthesis Kit (BIORAD, Hercules, CA, USA) according to manufacture instructions. Amplified cDNA was analyzed in triplicates using SsoFastTM EvaGreen® Supermix with Low ROX (BIO-RAD) with primers specific for either *SOX11*: 5’-GGTGGATAAGGATTTGGATTCG-3’ and 5’-GCTCCGGCGTGCAGTAGT-3’, or for the house-keeping gene *GAPDH*: 5’-AGTAGAGGCAGGGATGATG-3’ and 5’-TGGTATCGTGGAAGGACTC-3’.

### Western blot analysis of SOX11 and EZH2

8 x 10^6^ cells were harvested and protein extract preparation, quantification was performed as previously described by Gustavsson et al. [17]. Protein lysate (20 μg) were run on a NuPAGE 10% Bis-Tris gel (Invitrogen) and blotted on to a PVDF membrane using the iBLot® Dry Blotting System (Invitrogen). The membrane was blocked in 5% Milk/PBS before incubating with primary antibodies. Protein expression were assessed using the following antibodies: SOX11 monoclonal antibody [28], mouse anti GAPDH antibody (G8795, Sigma-Aldrich, St Louis, MO, USA) and EZH2 monoclonal antibody (Clone 11/EZH2, BD Transduction Laboratories, Franklin Lakes, NJ, USA). A HRP-labeled anti mouse antibody (P0260, DAKO, Glostrup, Denmark) was used for detection. Proteins were developed using SuperSignal West Femto Max Sensitivity Substrate (Pierce Biotechnology, Rockford, IL, USA) and images retrieved using a CCD-camera (Odyssey FC Imager from LI-COR Biosciences UK Ltd, Cambridge, England).

### Analysis of TCGA data

Level 2 methylation data from breast tumor samples from the TCGA data portal https://tcga-data.nci.nih.gov/tcga/ were processed as described for the human breast cells. Selecting unique female patients resulted in 669 tumors for further analysis. For 661 of the 669 samples, level 3 RNA sequencing data consisting of normalized gene counts was available. The transformation log2 (normalized gene count + 1) was used to generate gene expression levels for further analysis. Pearson correlation between corrected beta values and gene expression levels were used to investigate association between promoter methylation and gene expression levels. ER status was available for 599 of the tumors; 139 were ER-negative and 460 were ER-positive. Two-sided Wilcoxon tests were used to test for differences between ER-positive and ER-negative tumors.

### Histone ChIP

Chromatin immunoprecipitation of H3K27me3 and H3K4me3 bound regions were performed with the HighCell ChIP kit (Diagenode, Liege, Belgium) according to the protocol of the manufacturer. Antibodies against H3K27me3 (ab6002, Abcam, Cambridge, MA, USA) and rabbit IgG (Diagenode) were used in the ChIP experiments. Primers targeting the promoter of *GAPDH* (Diagenode) and *SOX11* (fwd: 5’-GAGAGCTTGGAAGCGGAGA-3’ rev: 5’-AGTCTGGGTCGCTCTCGTC-3’) were used.

### Treatment with Trichostatin A, Vorinostat and GSK343

Cells (1x10^6^) were seeded into a 6-well plate and cultured for 24 hours before drug treatment. Each cell line was treated with 0, 0.5 and 5 μM trichostatin A (Sigma-Aldrich), 0, 0.5 and 5 μM Vorinostat (Selleck, Houston, TX, USA) or 0, 10 and 20 μM GSK343 (Sigma-Aldrich ). For all treatments, DMSO was used as a vehicle control. After 24 h (Trichostatin A and Vorinostat) or 72 h (GSK343) of incubation, cells were harvested, protein lysate prepared and western blot performed as described above.

## Results

The aims of the present study were to explore the epigenetic regulation of *SOX11* in non-malignant and neoplastic cells of various origins and to assess the possibility to re-express SOX11 upon treatment with HDAC inhibitors.

### Epigenetic profiling of *SOX11* in non-malignant cells

The epigenetic regulation of *SOX11* in non-malignant cells has until now been widely unexplored. To assess if the previously observed *SOX11* promoter methylation and histone modifications in B cell lymphomas are a consequence of tumorgenesis or merely reflect the epigenetic status of the normal counterpart, non-malignant mature B cells from three differentiation stages, including naive, germinal center (GC) and memory B-cells, were FACS-sorted from tonsils (n=6).

**Figure 1:**
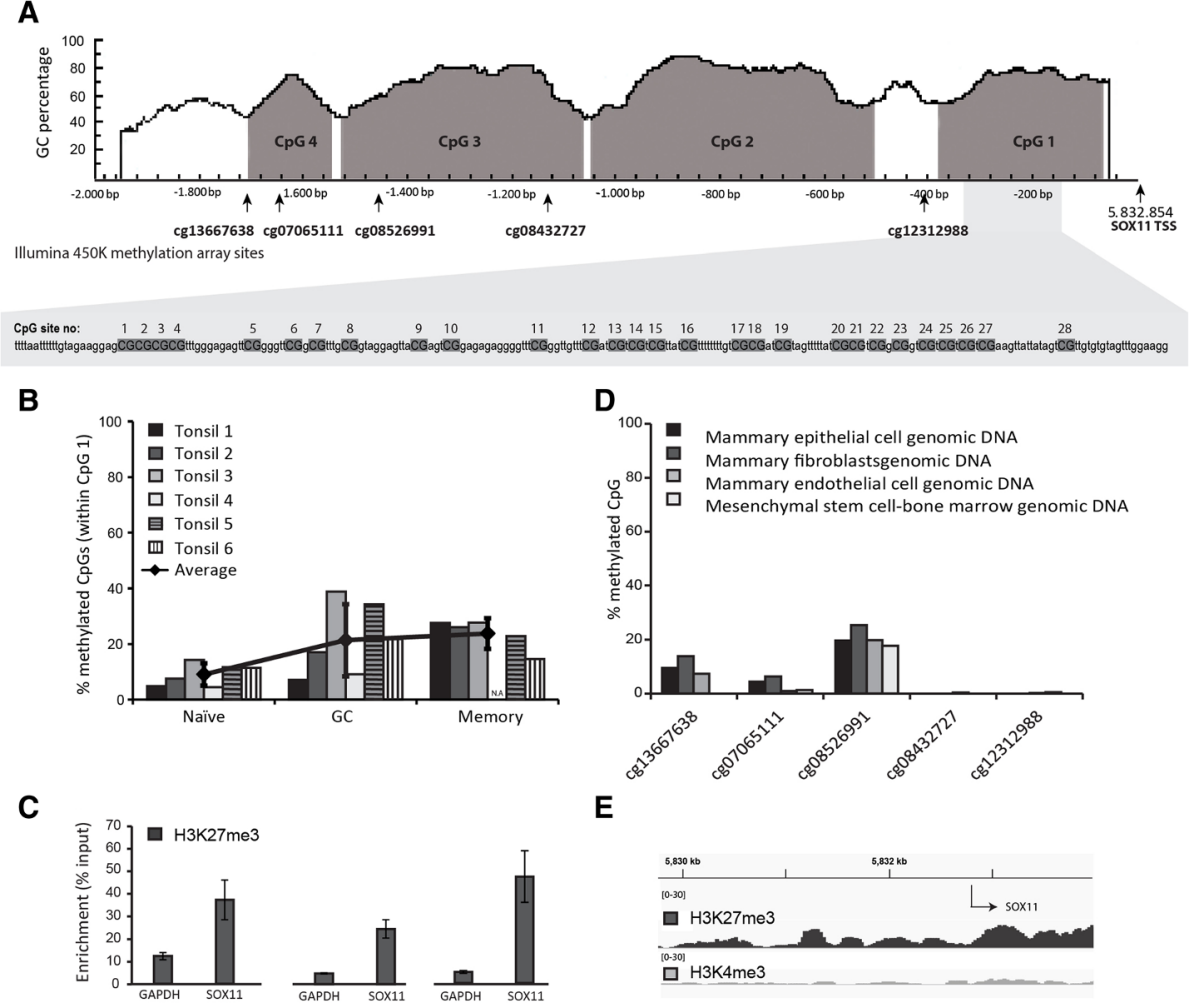
Epigenetic profiling of SOX11 in normal cells. **(A)** The SOX11 promoter 2000 bp upstream of transcription start site contains four CpG islands with analyzed CpG sites marked. **(B)** Mean *SOX11* promoter methylation within 28 CpG sites close to the transcription start site. **(C)** Enrichment of repressive H3K27me3, determined by ChIP-qPCR, within the *SOX11* promoter in naïve, GC and memory B-cells. **(D)** Methylation status of five CpG sites within the *SOX11* promoter, measured with Illumina 450 K methylation array, for several types of non-malignant mammary cell-types. **(E)** Enrichment of active (H3K4me3) and repressive (H3K27me3) histone marks in primary human mammary epithelial cells. ChIP-seq data was extracted from the ENCODE project.

The *SOX11* promoter contains four CpG islands, where the island most proximal to the transcription start site has been shown to be determinative for *SOX11* expression [17]. Consequently, 28 CpG sites within this CpG island were sequenced after bisulfite conversion (Figure 1). The fraction of methylated CpGs was calculated over all sequenced alleles (10-20 per sample) and revealed a low degree of *SOX11* methylation although a trend of increased methylation during differentiation was observed (Figure 1B). However, major inter-individual variations were observed, especially among the GC B cells. Using ChIP-qPCR, we further observed that the *SOX11* promoter showed a strong enrichment of H3K27me3 in all three B-cell populations (Figure 1C). As non-malignant reference tissue for solid tumors, DNA from human mammary fibroblasts, epithelial cells, and endothelial cells, as well as mesenchymal stem cells were analyzed on Illumina 450K methylation arrays (as part of a larger study). Information on five CpGs within the *SOX11* promoter was available. Analysis of the mammary cell types revealed that the two sites closest to the transcription start site were completely unmethylated while three sites upstream displayed a low degree of methylation (Figure 1D). Of note, cg12312988 is not located within a CpG island (see Additional file 2). Finally, ChIP-seq data (H3K27me3 and H3K4me3) on human mammary epithelial cells (downloaded from the ENCODE project [24]) showed strong enrichments of H3K27me3 over H3K4me3 on the *SOX11* promoter (Figure 1E).

### DNA methylation status of *SOX11* in lymphoid and solid tumors

**Figure 2:**
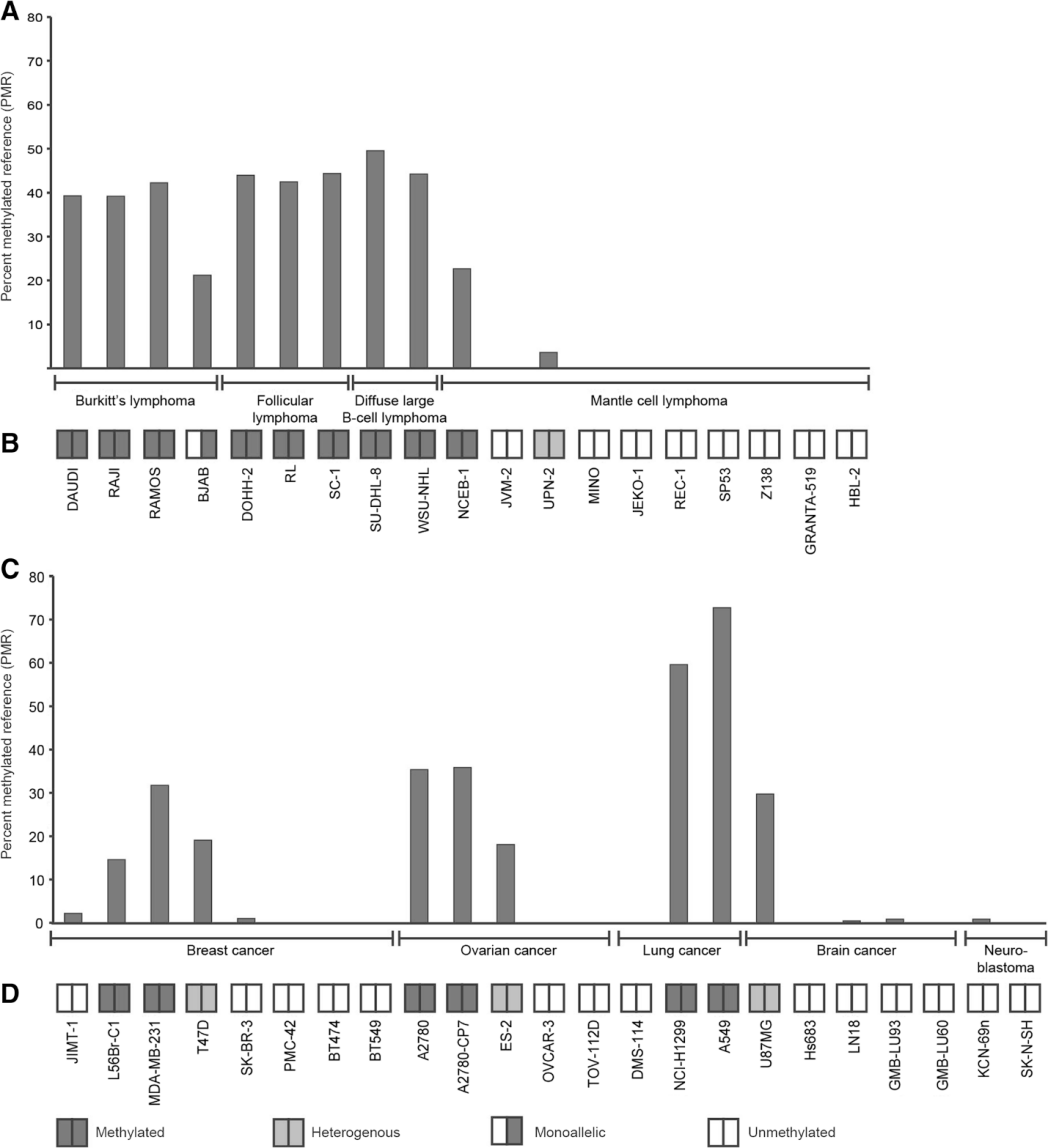
SOX11 promoter methylation in tumor cell-lines. *SOX11* promoter methylation status assessed with MethyLight and MS-MCA. The methylation levels analyzed by MethyLight are presented as percent methylated reference (PMR). PMR < 1 was considered as unmethylated promoter. *SOX11* promoter methylation was investigated in lymphoma cell lines with **(A)** MethyLight and **(B)** MS-MCA. *SOX11* promoter methylation was investigated in solid tumor cell lines with **(C)** MethyLight and **(D)** MS-MCA.

To explore the difference between non-malignant reference tissue and neoplastic cells, we further investigated the methylation status of *SOX11* in 42 cell lines (Table 1) representing a wide range of human tumors with subgroups known to express SOX11, including lymphoid malignancies (n=19), ovarian cancer (n=5), breast cancer (n=8), lung cancer (n=3), brain cancers (n=5) and neuroblastoma (n=2). To determine DNA methylation by complementary methods, MethyLight and methylation-specific melting curve analysis (MS-MCA) were used. The MethyLight and MS-MCA assays covered 8/28 and 28/28 CpG sites previously investigated in non-malignant mature B-cells, respectively (see Additional file 2). Overall, a good agreement between MethyLight and MS-MCA was observed in our sample set (Figure 2A and B), although calculated PMR values were significantly lower compared to absolute values derived from bisulfite sequencing of the same cell lines [17]. In agreement with public data [8,17,29], we show that *SOX11* is *de novo* methylated in all Burkitt’s lymphomas, follicular lymphomas and diffuse large B-cell lymphomas. In mantle cell lymphomas that express SOX11, the promoter is generally unmethylated (Figure 2A and B). Solid tumors show a much more diverse methylation pattern within the *SOX11* promoter (Figure 2C and D), possibly reflecting clinical subtypes with an altered epigenetic regulation.

**Table 1:** **Cell-lines investigated for SOX11 expression and promoter methylation**

Neoplasm	Cell-line
**Mantle cell lymphoma**	REC-1, GRANTA-519, JEKO-1, SP53, MINO, Z138, HBL-2, JVM-2, UPN-2, NCEB-1
**Follicular lymphoma**	DOHH-2, RL, SC-1
**Diffuse large B-cell lymphoma**	WSU-NHL, SU-DHL-8
**Burkitt’s lymphoma**	BJAB, RAJI, DAUDI, RAMOS
**Breast cancer**	JIMT-1, PMC-42, MDA-MB-231, SK-BR-3, T47D, BT474, BT9549, L56Br-C1
**Ovarian cancer**	OVCAR-3, TOV-112D, ES-2, A2780, A2780-CP7
**Lung cancer**	A549, DMS-114, NCI-H1299
**Glioma**	HS683, LN-18, U87MG, GMB-LU93, GMB-LU60
**Neuroblastoma**	SK-N-SH, KCN69n

### Correlation between promoter methylation and SOX11 expression

**Figure 3:**
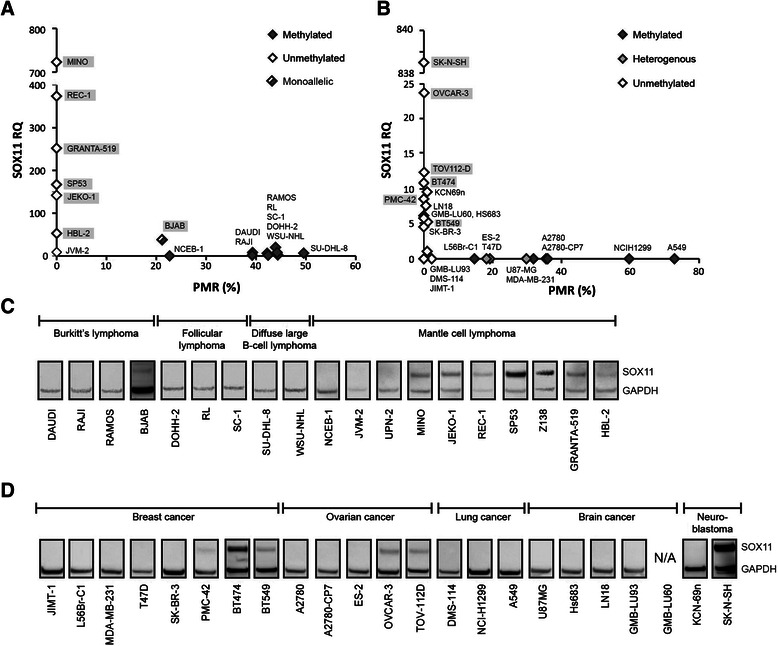
Correlation between *SOX11* promoter methylation and expression. The correlation between DNA methylation and gene expression was analyzed with RT-qPCR and western blot. In RT-qPCR, CT values >35 were considered below detection limit and corresponding *SOX11* levels were set to zero. **(A)** MethyLight (x-axis) and melt curve analysis (see filled, grey or open diamonds) showed an inverse correlation between *SOX11* mRNA and promoter methylation in lymphoma cell lines. **(B)** Likewise, inverse correlation between *SOX11* mRNA and promoter methylation was seen for solid cancer cell lines. For clarity, SOX11 positive (western blot) cell line names are shaded. **(C)** Western blot analysis of SOX11 and GAPDH in Burkitt’s lymphoma, follicular lymphoma, diffuse large B-cell lymphoma and mantle cell lymphoma cell-lines. **(D)** Western blot analysis of SOX11 and GAPDH in breast cancer, ovarian cancer, lung cancer, brain cancer and neuroblastoma cell lines.

**Figure 4:**
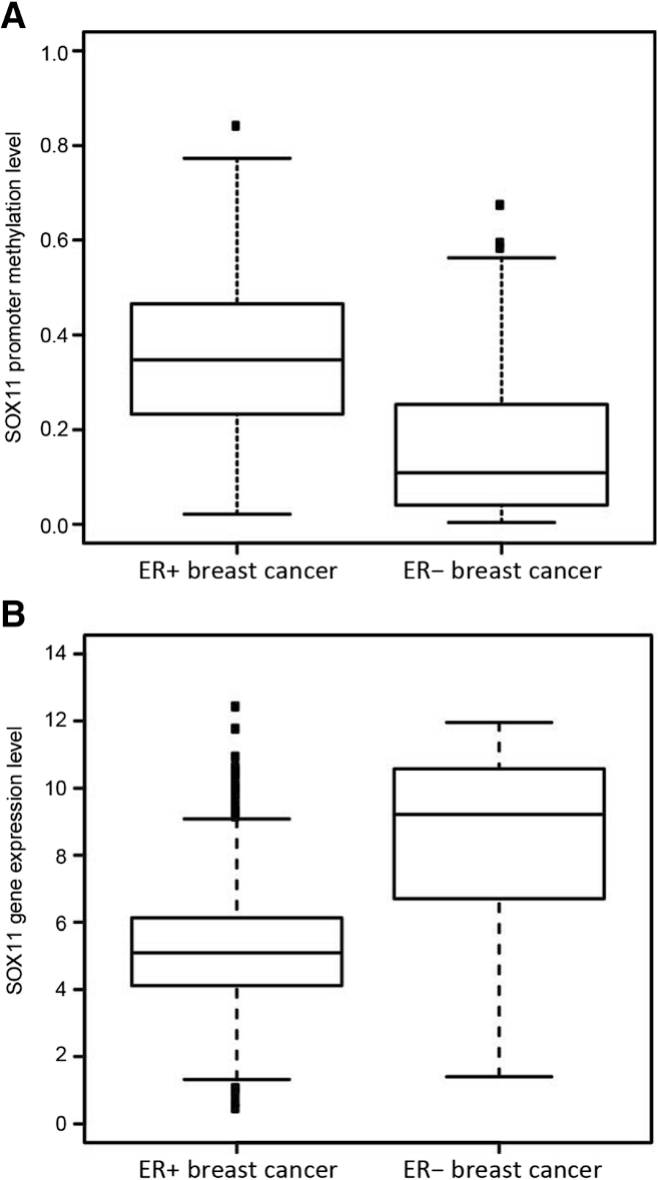
Correlation between SOX11 promoter methylation and ER positive breast cancer. **A)***SOX11* promoter methylation is significantly (p < 0.01) enriched in ER positive breast cancer (n = 460) compared to ER negative breast cancer (n = 139). **B)***SOX11* gene expression is significantly (p < 0.01) enriched in ER negative breast cancer compared to ER positive breast cancer.

To explore the correlation between *SOX11* promoter methylation and expression, each cell line was analyzed by RT-qPCR and western blot. An inverse correlation was observed between *SOX11* promoter methylation and gene expression for both lymphoid and solid tumor cells (Spearman’s correlation ρ=-0.71; p<0.001 and ρ=-0.75; p<0.001, respectively) (Figure 3A and B). SOX11 protein was detected in 7/8 (88%) MCL cell-lines with an unmethylated promoter (Figure 3C), while only 6/14 (43%) solid cancer cell lines with an unmethylated promoter had detectable levels of the protein (Figure 3D). As expected, none of the cell lines with a methylated promoter expressed *SOX11* mRNA or protein with the exception of BJAB. Using MS-MCA, we show that BJAB has a monoallelic methylation of the *SOX11* promoter, explaining the observed co-existence of a methylated promoter and expression of mRNA/protein (Figure 2A and Figure 3A and C). Pyrosequencing was further used to investigate if specific CpG sites are important for *SOX11* silencing in cell lines with low-to-intermediate methylation (as determined by MS-MCA and MethyLight). Data show that even at very low level of overall methylation, CpGs close to transcription start site are significantly methylated compared to expressing cell lines with a completely unmethylated promoter (Additional file 4). Finally, we demonstrate a correlation between *SOX11* methylation, expression and subtypes in primary breast cancers. Breast cancer methylation data and RNA-seq data were downloaded from The Cancer Genome Atlas (TCGA) and show that *SOX11* methylation is more abundant in estrogen recptor (ER) positive tumors (n=460) compared to ER negative tumors (n=139) (Figure 4A) with a strong anti-correlation between methylation and expression in each CpG site (Figure 4B and Table 2).

**Table 2:** **Correlation between** ***SOX11*** **promoter methylation and gene expression in primary breast cancers**

CpG site	Correlation	Std methylation
cg13667638	−0.40	0.24
cg07065111	−0.49	0.25
cg08526991	−0.50	0.26
cg08432727	−0.39	0.20
cg12312988	−0.16	0.12

Std gene expression = 2.42.

### Chromatin immunoprecipitation of H3K27me3 in neoplastic cells

**Figure 5:**
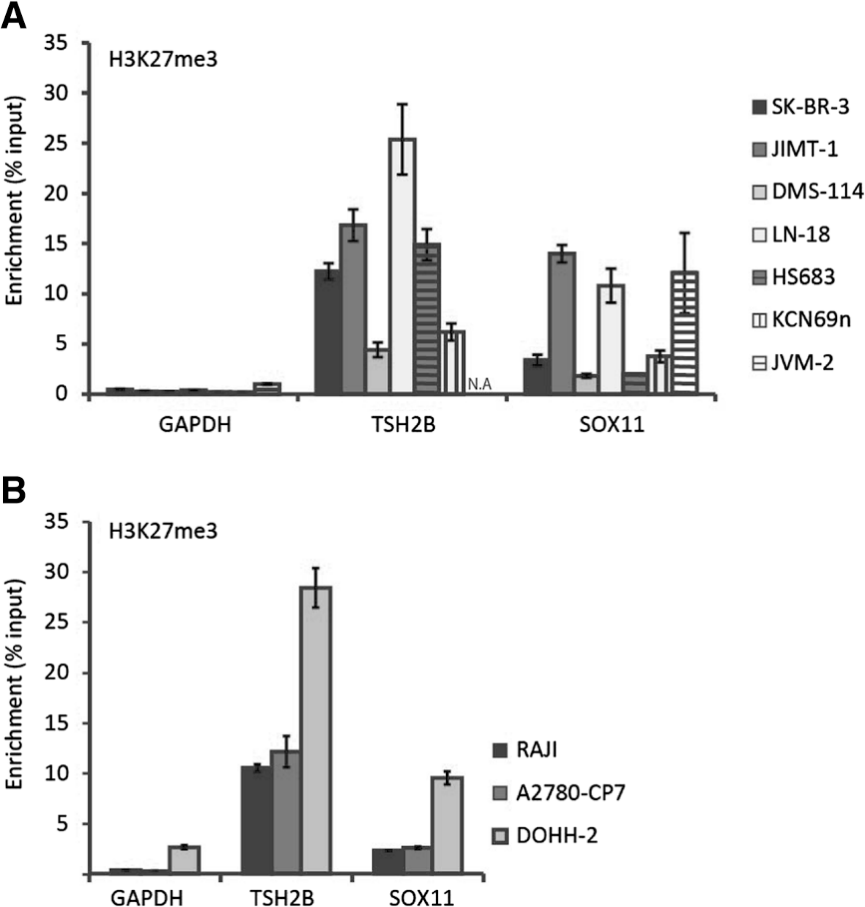
Enrichment of H3K27me3 within the *SOX11* promoter. Histone methylation of lysine 27 on histone 3 (H3K27me3) was assessed using chromatin immunoprecipitation and RT-qPCR for *GAPDH* (negative control), *TSH2B* (positive control) and *SOX11*. **(A)**Enrichment of H3K27me3 in unmethylated cell lines lacking SOX11. **(B)** Enrichment of H3K27me3 in methylated cell lines lacking SOX11.

As discussed above, normal cells have a strong enrichment of the silencing histone mark H3K27me3 on the promoter of *SOX11* but show a low degree of promoter methylation (Figure 1). In contrast, many neoplastic cell lines show a high degree of *SOX11* promoter methylation (Figure 3). To investigate if neoplastic cell lines with a low degree of methylation depend on H3K27me3 to silence *SOX11*, cell lines with a low or high degree of methylation were investigated to determine the enrichment of H3K27me3 at the *SOX11* promoter. The biological variation was significant, exemplified by the major variation in enrichment of the positive control, *TSH2B*. In two cell lines, DMS-114 and KCN-69n, the positive control showed such low levels of enrichment that data on *SOX11* cannot be interpreted. *GAPDH* was used as a negative control and background levels were set to the largest observed *GAPDH* value. We show that H3K27me3 at the *SOX11* promoter is enriched in several cell lines, including JIMT-1, LN-18 and JVM-2. However, SK-BR-3 and HS683 show a low enrichment compared to the positive control (*TSH2B*) and are likely dependent on other epigenetic regulation than promoter methylation or H3K27me3 to silence *SOX11* (Figure 5A). For comparison, three methylated cell-lines were analyzed and all three cell lines, DOHH-2, RAJI and A2780-CP7 showed low enrichment of H3K27me3 at the *SOX11* promoter compared to the positive control, indicating that methylation of the promoter may correlate to loss of repressive histone marks (Figure 5B).

### Trichostatin A and Vorinostat induce expression of SOX11 in unmethylated cells

**Figure 6:**
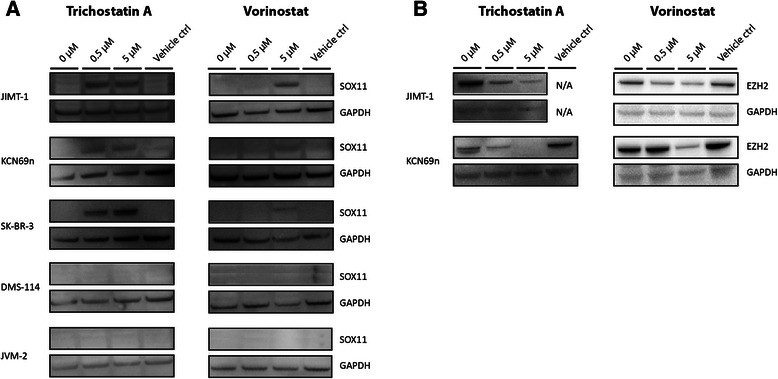
TSA and Vorinostat induce SOX11 expression and EZH2 down-regulation in unmethylated cancer cell-lines. Cells were treated with 0, 0.5 and 5 μM TSA or vorinostat for 24 hours followed by detection of SOX11 and EZH2 with western blot. **(A)** Unmethylated cancer cell lines JIMT-1, KCN69n and SK-BR-3 expressed SOX11 after treatment while DMS-114 and JVM-2 remained SOX11 negative. **(B)** JIMT-1 and KCN69n also showed a clear down-regulation of EZH2 upon treatment with HDAC inhibitors while the other cell lines did not show any change in EZH2 levels (data not shown).

Since the expression of SOX11 was shown to be regulated by repressive histone marks with or without an additional layer of methylation, we investigated the potential of two commonly used HDAC inhibitors vorinostat and trichostatin A (TSA), to re-express SOX11. The demethylating agent 5-aza-2’-deoxycytidine arrest proliferation already at low concentrations in lymphoid cells, and demethylation could thus not be assessed. Cell lines with no detectable levels of endogenous SOX11 with an unmethylated promoter were treated with 0, 0.5, and 5 μM of TSA or vorinostat for 24 hours. Both TSA and vorinostat induced SOX11 expression in SK-BR-3, JIMT-1 and KCN-69n. TSA was more potent than vorinostat, and showed protein induction already at 0.5 μM (Figure 6A). However, none of the drugs was able to induce SOX11 expression in DMS-114 and JVM-2, the latter in contrast to previous results [8]. Although some HDAC inhibitors have been reported to have a demethylating effect [30-32], we show that SOX11 expression could not be induced in any of the strongly methylated cell lines assessed, including RAJI, A2780-CP7 and DOHH-2 (Additional file 5A). Additionally, we demonstrate that EZH2, the enzyme responsible for H3K27 tri-methylation, was down-regulated upon TSA treatment (Figure 6B). To investigate if down-regulation of EZH2 is enough to induce expression of SOX11, the cell-lines were further treated with GSK343, an EZH2 inhibitor. However, despite EZH2 down-regulation in the majority of evaluated cell-lines, SOX11 was not re-expressed (Additional file 5B).

## Discussion

In non-malignant cells, epigenetic mechanisms are used to ensure flexible gene expression during development but later also permanent silencing of genes in differentiated tissues. Many human neoplasias display an altered epigenetic pattern, with overexpression or mutations of histone modifying enzymes and increased promoter methylation, leading to silencing of tumor suppressors [33]. These alterations are often reversible and the use of epigenetic drugs has become an attractive option to reprogram and sensitize cancer cells. During the last decade, both DNA demethylating agents (azacitidine and decitabine) and HDAC inhibitors (vorinostat and romidepsin) have been approved by FDA for use in myelodysplastic syndromes and cutaneous T-cell lymphoma, respectively [34-37]. Thus, epigenetic drugs have shown success in treatment of lymphoproliferative diseases, and several novel epigenetic drugs are currently in clinical trials for use in solid cancers.

With the growing interest in using epigenetic therapies in both hematological and solid malignancies, studies of novel epigenetically regulated genes are warranted and will provide (i) basic understanding, (ii) potential to use information on methylation in biomarker panels and (iii) opportunity to re-activate tumor suppressor functions or induce cancer stem cell differentiation [38-40] using novel epigenetic treatment strategies. We and others have during recent years shown that SOX11 is a diagnostic [13], prognostic [18,41,42], and functional biomarker in classical MCL [17], indolent MCL [43,44], ovarian cancer [15] and astrocytic gliomas [45]. SOX11 protein expression has been shown to correlate to increased and decreased survival in different tumor entities, emphasizing different function depending on molecular and cellular context.

Furthermore, initial epigenetic investigations shown that SOX11, which is a transcription factor normally expressed in a stage-specific manner during embryo development, has a bivalent histone mark (H3K4me3 and H3K27me3) [5]. Here we explore the relation between epigenetic regulation in non-malignant cells and neoplastic cells of various origin and demonstrate that non-malignant cells have a low degree of promoter methylation and are strongly marked by H3K27me3 in the *SOX11* promoter, independent on investigated cell lineage. Recently, several reports have suggested a crosstalk between DNA methylation and H3K27me3. It has been shown that several genes marked with H3K27me3 undergo *de novo* methylation in cancer [9]. In the B-cell lineage, Velichutina et al. observed that several EZH2 target genes involved in cellular growth, proliferation and differentiation become methylated in diffuse large B-cell lymphomas [46]. Additionally, Vire et al. demonstrated a physical interaction between DNA methyltransferases and EZH2 [47]. In agreement with this, SOX11 has been reported to be strongly methylated in most B-cell lymphomas [17], in nasopharyngeal carcinomas [19] and in bladder cancer [20]. This prompted us to further investigate the epigenetic regulation of *SOX11* in solid tumors.

Our data show that the pattern of *SOX11* methylation is more diverse within solid tumor types, compared to within B-cell lymphomas. Within each investigated tumor entity, *SOX11* could be unmethylated with or without protein expression or show a varying degree of methylation reflecting a large degree of inter-tumor heterogeneity. Interestingly, *SOX11* methylation correlates to ER positivity in breast cancer patients. The difference in epigenetic regulation related to breast cancer hormone status has previously been demonstrated by Müller et al. who showed difference in HDAC expression between ER positive and negative tumors [48]. In contrast to cell lines derived from solid tumors, B cell lymphoma cell lines show similar methylation pattern within each subtype of disease.

DNA microarray studies have shown that HDAC inhibitors induce selective changes in gene expression only affecting a small fraction of genes (2-10%) [49-51]. As SOX11 has shown to have a functional role and prognostic relevance in multiple cancer entities, we further investigated the potential to re-express SOX11 using epigenetic drugs. Using the HDAC inhibitors vorinostat and TSA, we show that SOX11 could be re-expressed in three out of five unmethylated cell lines but not in methylated cell lines, suggesting that promoter methylation protects the chromatin from being acetylated and the gene de-methylated and expressed. Furthermore, TSA and vorinostat treatment was shown to decrease the expression of EZH2 in cell lines that re-expressed SOX11, but not in others, further supporting an important role of EZH2 and H3K27me3 methylation in the maintenance of SOX11 silencing. Interestingly, Tiwari et al. recently demonstrated that SOX4, which share 91% sequence homology to SOX11 within the DNA binding domain [52], regulate the expression of EZH2 in mouse mammary epithelial and breast cancer cells [53]. However, using the EZH2 inhibitor GSK343, we show that decreased levels of EZH2 are not enough to re-express SOX11. Thus, as recently suggested by Helin et al. [54], H3K27me3 may be a passive mark of un-transcribed genes, and other epigenetic- or transcription factors may initiate the regulation. The re-expression using HDAC but not EZH2 inhibitors, demonstrate that, in addition to methylation and H3K27me3, also acetylation is important in the regulation of SOX11. Vorinostat and TSA inhibit a broad class of HDACs (HDAC1-4, HDAC6-7, and HDAC9) [55] and further investigations are needed to clarify which of these that control SOX11 expression. Although SOX11 was not re-expressed in methylated cell lines, an interaction between HDACs and CpG binding proteins has been demonstrated [56] and HDAC inhibitors have been reported to down regulate the expression of DNA methyl transferases [57].

## Conclusions

To assess the relation between epigenetic regulation of SOX11 in non-malignant tissue, lymphoid and solid malignancies, we investigated methylation and H3K27me3 enrichment at the *SOX11* promoter in populations of non-malignant B-cells and fibroblast cells compared to neoplastic cell lines of various origin. In non-malignant cells, *SOX11* is strongly marked by enrichment of H3K27me3 while tumors in general show promoter DNA methylation. Of interest, homogeneous methylation of the *SOX11* promoter is more frequently observed in lymphomas compared to solid tumors. Analysis of H3K27me3 enrichment in neoplastic cells show that cell lines with an unmethylated SOX11 promoter are strongly marked by H3K27me3, while methylated cell lines are associated with decreased H3K27me3 enrichment, indicating co-regulation of polycomb complex and DNA methyltransferases. We further show that down-regulation of EZH2 alone do not induce SOX11 expression but that clinically relevant HDAC inhibitors down-regulate EZH2 and induce SOX11 expression. Thus, H3K27me3 in combination with histone acetylation play an important role in SOX11 regulation, and emphasize the need to investigate the potential functional role of SOX11 upon epigenetic treatment and subsequent re-expression in patients with hematological or solid malignancies.

## Additional files

Additional file 1:
**Detailed description of cell lines, provider and culture media.**

Additional file 2:
**Overview of analyzed CpG sites with MS-MCA, MethyLight and pyrosequencing.**

Additional file 3:
**Interpretation of MS-MCA results.**

Additional file 4:
**Pyrosequencing of 28 CpG sites in low-to-medium methylated cell-lines.**

Additional file 5:
**Western blot showing treatment of SOX11 methylated cell-lines with TSA and SOX11 unmethylated and methylated cell-lines with GSK343.**
