# Supplementary material for: DNA methylation and histone modifications regulate SOX11 expression in lymphoid and solid cancer cells
Source: BMC Cancer. 2015 Apr 12;15:273. doi: 10.1186/s12885-015-1208-y (PMC4403777; doi:10.1186/s12885-015-1208-y)
Supplement: Additional file 1: — Detailed description of cell lines, provider and culture media. [file 12885_2015_1208_MOESM1_ESM.pdf]

Additional file 4 – Detailed description of cell lines, provider and culture media

Malignancy	Cell-line	Provider	Medium	FCS	L-glutamine	Other
MCL	REC-1	DSMZ	RPMI 1640	10 %	2 mM	
MCL	GRANTA-519	DSMZ	RPMI 1640	10 %	2 mM	
MCL	JEKO-1	DSMZ	RPMI 1640	20 %	2 mM	
MCL	SP53	1	RPMI 1640	10 %	2 mM	
MCL	MINO	DSMZ	RPMI 1640	20 %	2 mM	
MCL	Z138	2	RPMI 1640	10 %	2 mM	
MCL	HBL-2	N.A	RPMI 1640	10 %	2 mM	
MCL	JVM-2	DSMZ	RPMI 1640	10 %	2 mM	
MCL	UPN-2	N.A	RPMI 1640	10 %	2 mM	
FL	DOHH-2	DSMZ	RPMI 1640	10 %	2 mM	
FL	RL	DSMZ	RPMI 1640	10 %	2 mM	
FL	SC-1	DSMZ	RPMI 1640	10 %	2 mM	
DLBCL	WSU-NHL	DSMZ	RPMI 1640	10 %	2 mM	
DLBCL	SU-DHL-8	3	RPMI 1640	10 %	2 mM	
BL	BJAB	N.A	RPMI 1640	10 %	2 mM	
BL	RAMOS	DSMZ	RPMI 1640	10 %	2 mM	
BL	RAJI	DSMZ	RPMI 1640	10 %	2 mM	
BL	DAUDI	DSMZ	RPMI 1640	10 %	2 mM	
Ovarian	ES-2	4	McCoy's 5A	10 %	2 mM	
Ovarian	TOV-112D	4	DMEM high glucose	15 %	2 mM	
Ovarian	OVCAR-3	ATCC	RPMI 1640	20 %	2 mM	
Ovarian	A2780	5	RPMI 1640	10 %	2 mM	
Ovarian	A2780-CP7	5	RPMI 1640	10 %	2 mM	
Breast cancer	MCF7	ATCC	RPMI 1640	10 %	2 mM	
Breast cancer	JIMT-1	6	RPMI 1640	10 %	2 mM	
Breast cancer	SK-BR-3	ATCC	RPMI 1640	10 %	2 mM	
Breast cancer	PMC-42	6	RPMI 1640	10 %	2 mM	
Breast cancer	T-47D	ATCC	RPMI 1640	10 %	2 mM	
Breast cancer	MDA-MB-231	6	RPMI 1640	10 %	2 mM	
Breast cancer	L56Br-C1	6	RPMI 1640	10 %	2 mM	0.01 mg/ml insulin
Breast cancer	BT-474	7	RPMI 1640	10 %	2 mM	
Breast cancer	BT-549	7	RPMI 1640	10 %	2 mM	
Lung cancer	DMS-114	8	Waymouth's MB 752/1	10 %	2 mM	
Lung cancer	NCI-H1299	8	RPMI 1640	10 %	2 mM	
Lung cancer	A549	8	Ham's F12 Kaighn's	10 %	2 mM	
Glioma	HS683	9	DMEM high glucose	10 %	4 mM	1 mM sodium pyruvate
Glioma	LN18	10	DMEM high glucose	10 %	2 mM	
Glioma	U-87 MG	10	DMEM high glucose	10 %	2 mM	
Glioma	GMB-LU60	11	RPMI 1640	10 %	2 mM	1 mM Sodium Pyruvate, 10 mM HEPES, 50 ug/ml gentamicin
Glioma	GMB-LU93	11	RPMI 1640	10 %	2 mM	1 mM Sodium Pyruvate, 10 mM HEPES, 50 ug/ml gentamicin
Neuroblastoma	KCN-69n	12	MEM/EBSS	10 %	2 mM	
Neuroblastoma	SK-N-SH	12	MEM/EBSS	10 %	2 mM	

Cell-lines were kindly provided by:

1. Mats Ehinger, Dept. of Pathology, University Hospital Lund, Sweden
2. Martin Dyer, University of Leicester, Leicester, England
3. Kristina Drott, Division of Hematology and Transfusion Medicine, Lund University, Sweden
4. Gabriella Honeth, Department of Cell and Molecular Biology, Section for Tumor Biology, Lund University, Sweden

5. Donal J Brennan, UCD School of Biomolecular and Biomedical Science, UCD Conway Institute, University College, Dublin, Ireland
6. Cecilia Hegardt, Department of oncology, Lund University, Sweden
7. Sofia Gruvberger Saal, Department of Oncology, Lund University, Sweden
8. BioInvent International AB, Lund, Sweden
9. Ulrike Nuber, Department of Laboratory Medicine, Lund Strategic Research Center for Stem Cell Biology and Cell Therapy, Lund University, Sweden
10. Mattias Belting, Department of clinical Science, Section of oncology, Lund University, Sweden
11. Anna Darabi, Department of Clinical Sciences, Division of Neurosurgery, Lund University, Sweden
12. Sven Påhlman, Department of Laboratory Medicine, Lund University, Sweden
