# Supplementary figures and images for: DNA methylation and histone modifications regulate SOX11 expression in lymphoid and solid cancer cells
Source: BMC Cancer. 2015 Apr 12;15:273. doi: 10.1186/s12885-015-1208-y (PMC4403777; doi:10.1186/s12885-015-1208-y)

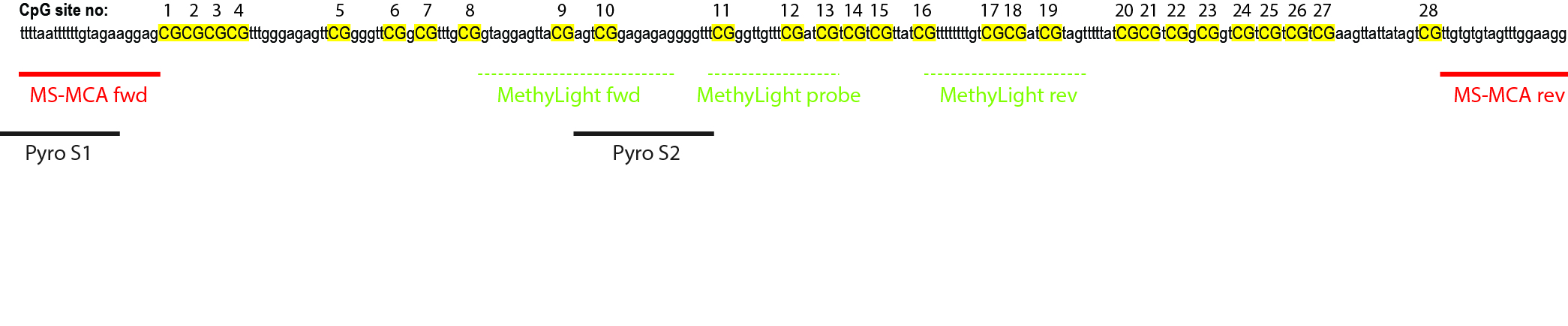

Supplement: Additional file 2: — Overview of analyzed CpG sites with MS-MCA, MethyLight and pyrosequencing. [file 12885_2015_1208_MOESM2_ESM.jpeg]

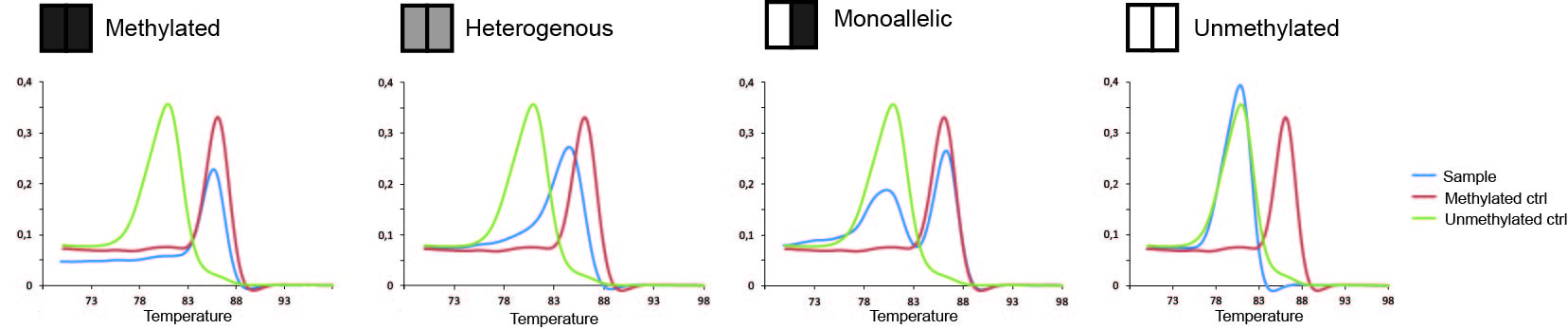

Supplement: Additional file 3: — Interpretation of MS-MCA results. [file 12885_2015_1208_MOESM3_ESM.jpeg]

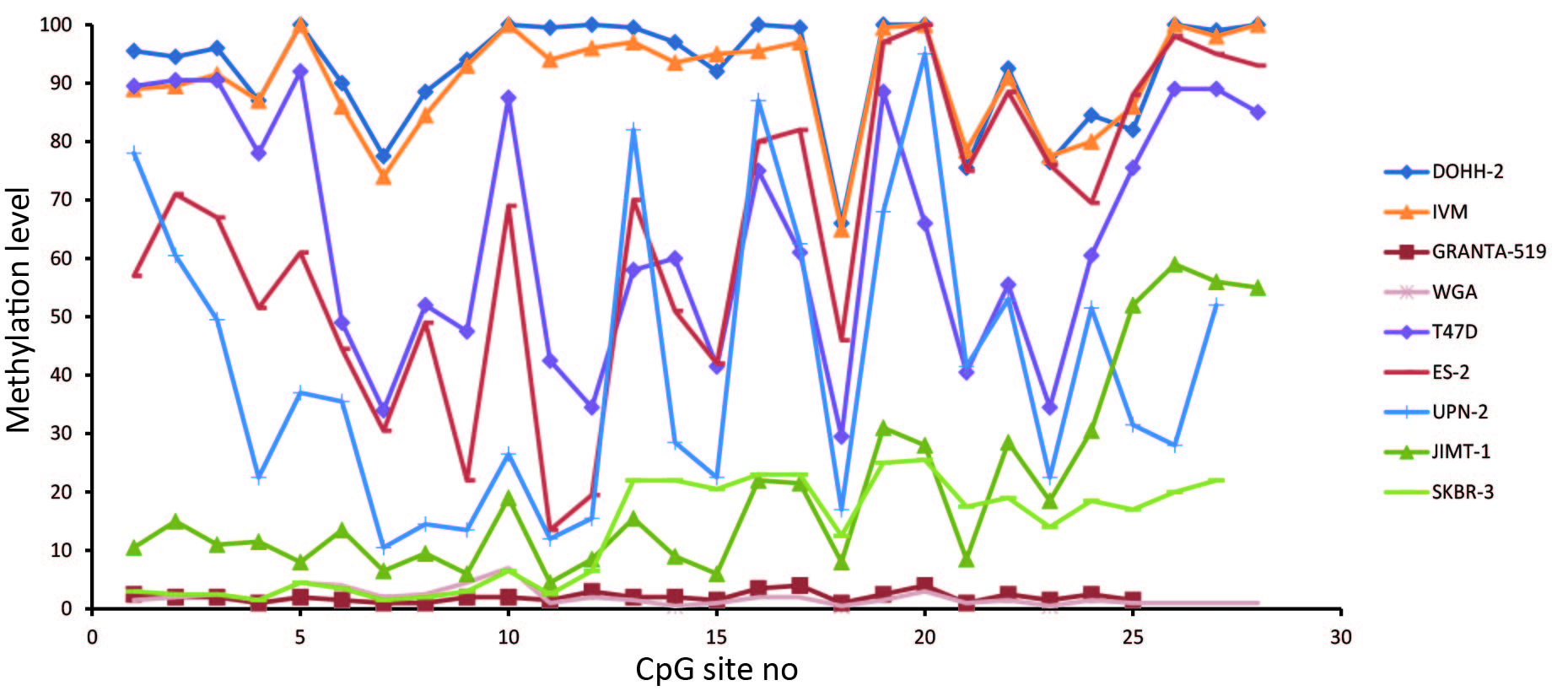

Supplement: Additional file 4: — Pyrosequencing of 28 CpG sites in low-to-medium methylated cell-lines. [file 12885_2015_1208_MOESM4_ESM.jpeg]

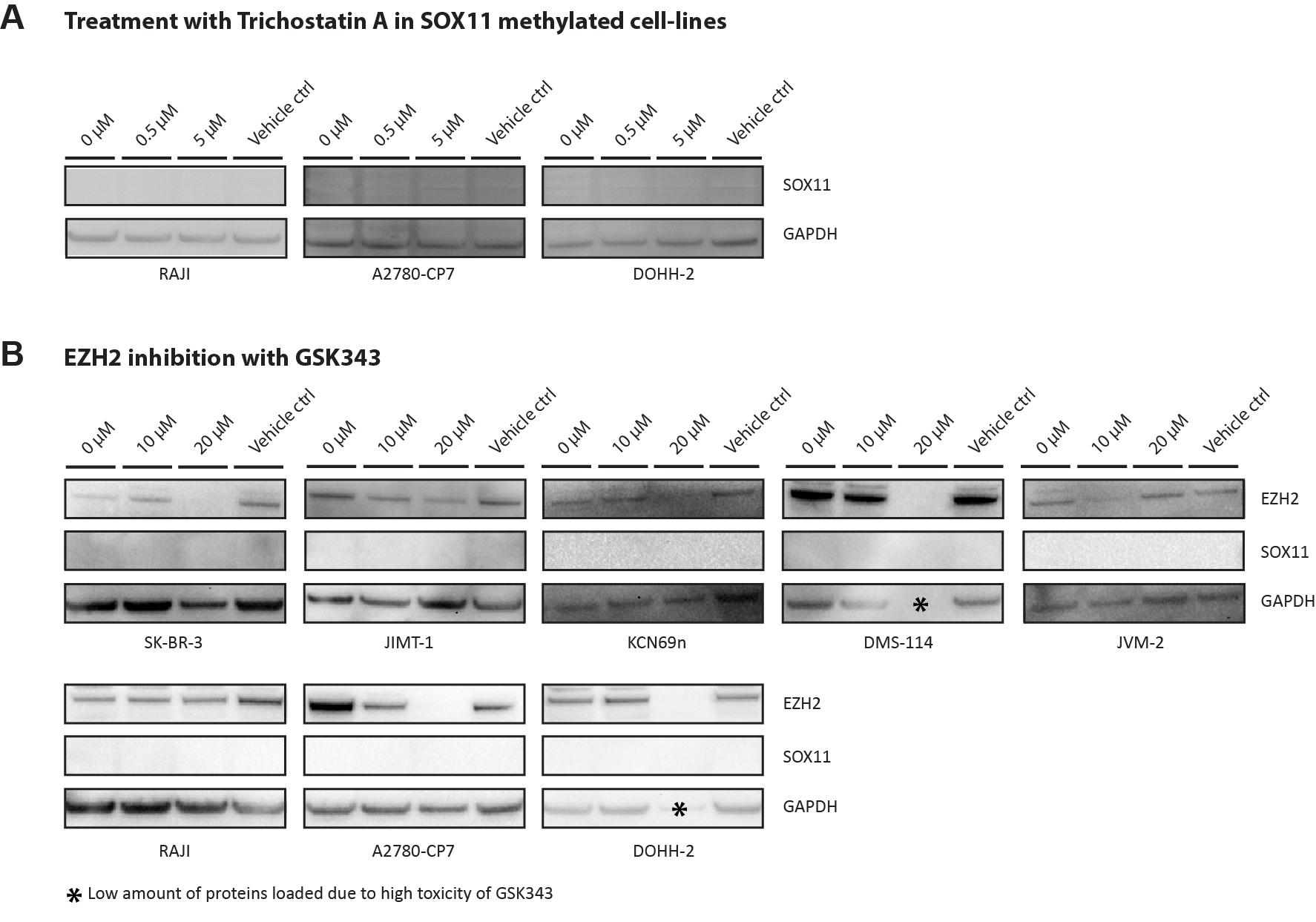

Supplement: Additional file 5: — Western blot showing treatment of SOX11 methylated cell-lines with TSA and SOX11 unmethylated and methylated cell-lines with GSK343. [file 12885_2015_1208_MOESM5_ESM.jpeg]
